# Relationships between Sclerostin, Leptin and Metabolic Parameters in Non-Dialysis Chronic Kidney Disease Males

**DOI:** 10.3390/jpm13010031

**Published:** 2022-12-23

**Authors:** Katarzyna Romejko, Aleksandra Rymarz, Katarzyna Szamotulska, Zbigniew Bartoszewicz, Stanisław Niemczyk

**Affiliations:** 1 Department of Internal Diseases, Nephrology and Dialysis, Military Institute of Medicine, 04-141 Warsaw, Poland; 2 Department of Epidemiology and Biostatistics, Institute of Mother and Child, 01-211 Warsaw, Poland; 3 Department of Internal Diseases and Endocrinology, Medical University of Warsaw, 02-097 Warsaw, Poland

**Keywords:** chronic kidney disease, sclerostin, leptin, metabolic parameters

## Abstract

Sclerostin is an inhibitor of the Wnt-beta-catenin pathway. The relationship between sclerostin and adipose tissue or between sclerostin and nutritional status has been the subject of research interest in the last decade. Sclerostin concentrations are elevated in patients with chronic kidney disease (CKD). Leptin is an adipocytokine which inhibits food intake by stimulating the satiety center in the hypothalamus. Leptin concentrations rise with the reduction of eGFR (glomerular filtration rate). The aim of this study was to investigate the possible association between sclerostin and leptin, between sclerostin and selected poor prognostic factors of CKD progression, and between sclerostin and nutritional parameters in non-dialysis CKD male patients. 101 men with non-dialysis CKD stage 3–5 were included in the study. Bioimpedance spectroscopy (BIS) was used to measure body composition. Blood samples were drawn to measure the serum concentrations of sclerostin, leptin, creatinine, hemoglobin (Hgb), parathormone (PTH), inflammatory markers, and markers of nutritional status. We also measured homeostatic model assessment of insulin resistance (HOMA-IR) as well as blood pressure. We observed a significant, positive relationship between sclerostin and age, leptin, and glycated hemoglobin (HgbA1c) concentrations. A significant, negative association was observed between sclerostin and eGFR. Sclerostin is associated with leptin in non-dialysis CKD male patients. Sclerostin is also related to metabolic disturbances such as hyperglycemia in this population.

## 1. Introduction

Chronic kidney disease (CKD) is one of the major public health problems. Almost 13% of the world’s population suffer from irreversible kidney damage [1]. Hyperlipidemia, overhydration, metabolic, endocrine, and mineral bone disorders along with anemia, malnutrition, and electrolyte disturbances are frequent complications of CKD [2,3].

For a long time, adipose tissue has been thought to be mainly connected with thermoregulation. Over the last two decades it has been proven that adipose tissue is an endocrine organ which synthesizes and secretes various hormones. Adipocytokines communicate with other tissues and organs and affect metabolic balance. The concentration changes of adipocytokines have an influence on the development of cardiovascular disease, atherosclerosis, hypertension, and diabetes and may also have an impact on nutritional and inflammatory status [4,5]. One of adipose tissue hormones is leptin. It is produced mainly by adipocytes but is also synthesized in brain, skeletal muscles, placenta, and intestines [6,7]. Leptin inhibits food intake by stimulating the satiety center in the hypothalamus [8]. It also increases energy expenditure and stimulates the production of inflammatory cytokines such as tumor necrosis factor-alpha (TNF-alpha), interleukin-6 (IL-6), and interleukin-12 (IL-12) [9,10]. Leptin concentrations increase in CKD as a result of decreased elimination through the kidneys due to reduced glomerular filtration rate (eGFR) and the impairment of leptin metabolic degradation in the renal tubules [11]. Some studies proved that leptin participates in the development of malnutrition in CKD, but further investigations are needed to comprehend the exact mechanisms of weight loss in this group of patients [12].

Sclerostin is mainly synthesized by osteocytes, but the expression of sclerostin mRNA was also observed in other tissues such as kidney, liver, pancreas, lung, heart, and vascular muscle cells [13]. Wnt are the large family of secreted glycoproteins which play a role in embryogenic development and tissue generation [14]. Sclerostin negatively regulates the Wnt-beta-catenin pathway which results in the inhibition of bone formation, especially by affecting osteoblasts function, lowering their proliferation and differentiation. Sclerostin also enables the apoptosis of osteoblasts and may have a positive effect on osteoclast formation and maturation [15]. Sclerostin concentrations increase with the development of CKD and are almost threefold higher in patients with end-stage renal disease than in individuals without renal failure [16]. Successful renal transplantation and the improvement of kidney function result in the decrease of sclerostin levels [17]. Sclerostin concentrations are observed to be higher in elderly individuals and also increase with age in CKD patients [18,19,20]. It was reported that sclerostin concentration is elevated in diabetes patients independently of eGFR [20,21]. Sclerostin also intensifies ectopic calcification [22]. Sclerostin levels are associated with vascular calcification in patients with CKD. Vascular calcification in this group of patients contributes to increased morbidity and mortality [23,24].

Malnutrition is a common finding in CKD patients [25]. Appetite disorders in patients with CKD lead to the loss of body protein resources. High inflammatory cytokine levels, hyperinsulinemia and insulin resistance, metabolic acidosis, resistance to growth hormone, and hyperparathyroidism as well as depression and low economic status may also lead to malnutrition in CKD patients [26,27]. Additionally, hormonal disorders, including high leptin concentrations, play a role in the development of malnutrition in CKD patients [28].

The relationship between adipocytokines, nutritional status and sclerostin has been the subject of research interest of the last decade. Some reports reveal that there may be a connection between sclerostin and adipose tissue or between sclerostin and nutritional status, but others do not show this association [29,30,31,32].

The purpose of this study was to investigate the possible association between sclerostin concentrations with leptin, between sclerostin and selected poor prognostic factors of CKD progression such as hyperglycemia, insulin resistance, anemia, and high inflammatory status, and between sclerostin and nutritional parameters in non-dialysis CKD male patients.

## 2. Materials and Methods

### 2.1. Design

An observational cross-sectional study in non-dialysis CKD male was performed. The inclusion criterion was eGFR lower than 60 mL/min/1.73 m².

### 2.2. Patients

The study sample consisted of 101 men with non-dialysis CKD and eGFR lower than 60 mL/min/1.73 m² recruited to the study between November 2018 and February 2020. Patients visited Nephrological Outpatient Clinic of Military Institute of Medicine in Warsaw, Poland, for a routine check-up. If they agreed to participate in the study and fulfilled the inclusion criteria of eGFR <60 mL/min/1.73 m² and age 18–80 years, a new visit was arranged. Patients were classified into different levels of CKD according to KDIGO 2012 Clinical Practice Guideline for the Evaluation and Management of Chronic Kidney Disease. Renal replacement therapy or its requirement within the following 3 months, clinical signs of infection, the presence of metal parts in the body, eGFR ≥60 mL/min/1.73 m², and the lack of agreement to participate in the study were the exclusion criteria. Additionally, patients who were treated with ESA (Erythropoietin Stimulating Agent) were not qualified for the study. The day before the examination, participants were asked to avoid physical exercise and alcohol consumption. All participants signed an informed consent. The local ethics committee accepted the study protocol (Bioethics Committee in Military Institute of Medicine in Warsaw, Poland, IRB acceptance number 120/WIM/2018 obtained 22 August, 2018).

Blood samples were drawn after an overnight fast. Serum creatinine concentrations were measured using the Jaffe method (Gen.2; Roche Diagnostics GmbH, Risch-Rotkreuz, Switzerland). Serum albumin levels were measured using BCP Albumin Assay Kit (Roche Diagnostics GmbH, Risch-Rotkreuz, Switzerland). Samples for measuring sclerostin and leptin levels were kept frozen at −80 °C. SOST, leptin, and TNF-alpha levels were assessed using the Bio-Plex MAGPIX (Luminex Corporation, Austin, TX, USA).

Body composition was measured by bioimpedance spectroscopy with the use of a Body Composition Monitor (Fresenius Medical Care, Bad Homburg, Germany). Patients remained in a supine position after a five-minute rest and electrodes were placed in a tetrapolar configuration (on one hand and one foot).

eGFR was calculated according to the short Modification of Diet in Renal Disease (MDRD) formula [29]. GFR in mL/min per 1.73 m² = 175 × SerumCr − 1.154 × age − 0.203 × 1.212 (if patient is black) × 0.742 (if female).

### 2.3. Defining the Nutritional Parameters

The nutritional parameters were defined as follows:

- serum concentrations of albumin
- total cholesterol serum level
- body mass index (BMI)
- relative fat (Rel FAT)
- relative lean tissue mass (Rel LTM)

### 2.4. Statistical Analysis

The results are presented as means ± standard deviations (SD) for normally distributed data, or medians and interquartile ranges (IQR) for non-normally distributed variables. The Kolmogorov-Smirnov test was used for evaluating distributions for normality. For gradual changes, estimation across categories one-way ANOVA with linear trend analysis or Jonckheere-Terpstra test for trend were applied, where appropriate. Bivariate associations between continuous variables were assessed by Spearman’s rho. For controlling confounders, stepwise multivariate quantile regression was used. A *p*-value < 0.05 was considered to be statistically significant. Statistical analysis was performed using Stata Statistical Software: Release 17 (StataCorp. 2021, College Station, TX, USA: StataCorp LLC).

## 3. Results

The study sample consisted of 101 non-dialysis CKD male patients with eGFR lower than 60 mL/min/1.73 m². The median age of participants was 66 years (59–72). There were 37 patients in the G3A group (eGFR 45–59 mL/min/1.73 m²), 32 in the G3B group (eGFR 30–44 mL/min/1.73 m²), and 32 in G4-G5 group (eGFR ≤ 29 mL/min/1.73 m²). Median sclerostin concentration was 373.74 pg/mL and progressed with the advancement of CKD (*p* = 0.015). With the severity of kidney function degradation, we found statistically significant changes of nutritional and metabolic parameters. We reported a reduction in the concentrations of albumin (*p* < 0.001), low-density lipoprotein cholesterol (LDL-C) (*p* = 0.017), hemoglobin (Hgb) (*p* < 0.001), hematocrit (Hct) (*p* < 0.001), and the increase of fibrinogen (*p* = 0.012), TNF-alpha (*p* < 0.001), parathormone (PTH) (*p* < 0.001), and glucose (*p* = 0.016) levels. Also, the median values of glycated hemoglobin (HgbA1c) were higher in CKD 3B and CKD 4–5 than in CKD 3A. However, the trend analysis did not confirm the association of HgbA1c with CKD stage (*p* = 0.056) (Table 1).

We observed a statistically significant correlation between sclerostin concentrations and eGFR (*p* = 0.004) and between sclerostin concentrations and age (*p* = 0.006). Sclerostin concentrations were higher in patients with lower eGFR and in elderly individuals (Table 2). Serum sclerostin concentrations correlated positively with glucose level (*p* = 0.005), HgbA1c (*p* < 0.001), fibrinogen (*p* = 0.021), leptin (*p* = 0.001), and PTH (*p* = 0.014). We found a statistically significant, negative correlation between sclerostin concentrations and Rel LTM (*p* = 0.017), Hgb (*p* = 0.001), and Hct (*p* = 0.001) (Table 2).

Variables significantly associated with sclerostin in correlation analysis (Table 2) were chosen for the stepwise multivariate quantile regression. In the estimated model, HgbA1c, leptin, and age remained statistically significant after adjustment for eGFR (*p* < 0.001, *p* = 0.003, *p* < 0.001 respectively, Table 3).

Parallelly, leptin concentrations were inversely correlated with Rel LTM (*p* < 0.001) and positively correlated with homeostasis model assessment of insulin resistance (HOMA-IR) (*p* < 0.001), inflammatory markers such as CRP (C-reactive protein) (*p* = 0.038), fibrinogen (*p* = 0.004) and TNF-alpha (*p* = 0.008), Rel FAT (*p* < 0.001), and BMI (body mass index) (*p* < 0.001) and weight (*p* < 0.001) (Table 4).

## 4. Discussion

The relationship between sclerostin and nutritional status has been the subject of research interest in the last decade. The report of Kim proved that sclerostin has a significant negative association with skeletal muscle mass [33]. Hemodialysis patients with elevated serum sclerostin levels also had lower muscle mass [34]. There are studies which suggest that sclerostin may also play an endocrine function and communicate between skeletal and adipose tissue [31]. The mechanisms of communication between sclerostin and adipose tissue are not entirely understood and require further investigation. In our study we found a relationship between sclerostin and adipocytokine-leptin in CKD male patients.

In our report, we confirmed that sclerostin concentrations rise in the groups of patients with more advanced CKD. Additionally, a significant inverse correlation between sclerostin concentrations and eGFR was observed. Sclerostin levels also increased with age and were positively related with HgbA1c. Moreover, we found an association between sclerostin and leptin, a molecule which is known to be an anorexigenic factor.

According to other investigators, the rise of sclerostin in CKD patients was thought to be caused by the reduction of GFR, but the study of 120 patients with CKD proved that increased sclerostin serum levels did not result from decreased renal elimination because urinary excretion of sclerostin increased with the declining renal function [35]. There are also several other hypotheses of high sclerostin concentrations in patients with CKD. Elevated sclerostin serum concentrations in CKD may be due to its increased production. Sabbagh examined the progression of renal osteodystrophy in jck mice. With the development of CKD in mice, the progression of osteoclast activity was observed. The repression of Wnt-beta-catenin signalling within osteocytes and increased expression of Wnt-antagonists such as sclerostin was found [36]. Nevertheless, the causes of increased expression of sclerostin in CKD are not yet well known. On the other hand, the treatment with sevelamer-HCl which decreases phosphate overload in CKD led to a significant decrease in serum sclerostin concentration which indicates that high phosphate concentrations in CKD may increase sclerostin levels [37]. Additionally, Bonani showed that sclerostin concentrations decrease rapidly after a successful renal transplantation and improvement of renal function [17].

Sclerostin levels rise with age [18]. The concentrations of sclerostin increase with age in non-dialysis CKD patients and correlate positively with age in HD patients [19,20]. We also observed a positive, significant association between sclerostin concentrations and age in the studied population, which was confirmed in multivariate analysis.

Some studies proved that sclerostin concentrations are higher in diabetic patients, with and without chronic kidney disease, in comparison with non-diabetic individuals [20,21]. In our study we analyzed poor prognostic factors of CKD progression. Positive, significant correlation was observed between sclerostin concentrations and HgbA1c. Sclerostin concentrations were significantly higher in patients with elevated HgbA1c. We may conclude that sclerostin is a possible marker of metabolic disturbances such as hyperglycemia in non-dialysis CKD male patients. Sclerostin may also play a role in the intensification of metabolic derangements in the studied population.

As was previously mentioned, hormonal disorders such as high leptin concentrations are thought to play a role in the development of malnutrition in CKD. Leptin levels rise with the reduction of eGFR due to its lower glomerular filtration and decreased metabolic degradation in the renal tubules. In our report, leptin was significantly associated with elevated levels of inflammatory cytokines such as CRP, fibrinogen, and TNF-alpha. Additionally, high leptin concentrations were also significantly associated with increased insulin resistance assessed by HOMA-IR. Insulin resistance also takes part in the decrease of muscle mass in CKD patients. In our report, leptin correlated negatively with Rel LTM which means that high leptin levels were related with lower muscle mass. We may conclude that increased leptin concentrations in CKD male patients may lead to decreased muscle mass by elevating inflammatory status and insulin resistance. In our study, we observed a positive association between sclerostin and leptin in non-dialysis CKD male patients. The association between sclerostin and an anorexigenic hormone such as leptin may indicate that sclerostin plays a role as a risk factor of developing malnutrition in CKD patients. We may also assume that high sclerostin concentrations in CKD may favor the elevation of leptin levels. Additionally, as was previously mentioned, searching for a possible connection between sclerostin and adipose tissue, and between sclerostin and nutritional status, has been the subject of numerous studies in the last decade [29,30,31,32]. The results of our study showed an association between sclerostin and leptin in non-dialysis CKD patients. In our univariate analysis, SOST concentrations were significantly higher in patients with lower Rel LTM and in those with Hgb < 11 g/dL. These findings were not confirmed in a multivariate model where sclerostin was associated significantly with HgbA1c ≥ 6.5%, leptin, and age. A possible reason for a lack of significant association between sclerostin and Rel LTM, and between sclerostin and Hgb in multivariate analysis, is that Rel LTM negatively relates to leptin. High leptin concentrations can cause more severe metabolic disorders which may lead to a reduction in muscle mass. Moreover, the lack of significant association between sclerostin and Rel LTM, and between sclerostin and Hgb in multivariate analysis, and the presence of these relationships in univariate analysis does not preclude that there is a possible confounding effect of leptin in the relationship between sclerostin and decreased muscle mass presented as lower Rel LTM, and between sclerostin and anemia in non-dialysis CKD male patients.

In our study we found that the concentrations of other nutritional parameters such as albumin and cholesterol change with the progression of kidney function decrease. Serum albumin concentrations decreased with the reduction of eGFR and LDL-C. The level of total cholesterol (TC) also decreased with the development of CKD; however, its reduction was at the border of significance (Table 1). The correlations between sclerostin and nutritional parameters such as albumin and total cholesterol did not reach statistical significance, but we found that albumin, total cholesterol (TC), LDL-C, and high-density lipoprotein cholesterol (HDL-C) concentrations were reduced in patients with elevated sclerostin levels (Table 2). Despite the lack of statistical significance of these associations, their negative direction may suggest that increased sclerostin levels can be a possible cause of nutritional derangements and the development of malnutrition in the population of non-dialysis CKD patients.

Some studies show the association between sclerostin and PTH concentrations. In peritoneal-dialysis patients and in HD patients, PTH concentrations correlated negatively with serum sclerostin concentrations [26,38]. It was proved that low PTH concentrations induce sclerostin production and that PTH directly inhibits the transcription of SOST gene in vivo and in vitro, which suggests that SOST regulation may play a role in mediating PTH action in bone [39]. In our report, we did not find the correlation between sclerostin and PTH in multivariate analysis.

The study of Catalano proved that, in the group of patients with type 1 diabetes mellitus, there is a difference in serum sclerostin concentrations between women and men. Women with type 1 diabetes mellitus had higher circulating sclerostin levels compared to men [40]. In our study, we included the group of men only and therefore we could not compare the results according to gender. New studies with CKD participants are needed to evaluate if sclerostin serum concentrations differ between men and women in this group of patients.

Our design is not without limitations. This is a cross-sectional study. The sample size was relatively small. Larger groups of participants would allow for dividing the population into subgroups according to stages of CKD and to examine if the analyzed complications are more severe with the decrease of eGFR. Another limitation of this study is that our report does not consider the effects of treatment, especially antihypertensive drugs and renal anemia therapy.

## 5. Conclusions

The relationship between sclerostin, adipose tissue, and nutritional derangements are the subject of new reports. In our study, we reported that sclerostin is associated with leptin in non-dialysis CKD male patients. Sclerostin is also related with metabolic disturbances such as hyperglycemia in the studied population. Further studies are needed to look for the relationship between sclerostin, adipose tissue, and nutritional status in CKD in terms of possible future preventive and therapeutic procedures.

## Figures and Tables

**Table 1:** Clinical characteristics of the study population.

Characteristic	*n*	Total (*n* = 101)	CKD 3A (*n* = 37)	CKD 3B (*n* = 32)	CKD 4–5 (*n* = 32)	*p*_trend_-Value
Sclerostin (pg/mL)	101	373.74 (266.32–579.73)	318.88 (221.87–505.88)	392.87 (267.71–547.65)	446.29 (313.7–679.61)	**0.015**
Age (years)	101	66 (59–71)	67 (57–72)	70 (65–75)	61 (54–68)	0.176
Weight (kg)	98	89.3 ± 16.2	90.9 ± 13.7	86.8 ± 16.9	90.1 ± 18.3	0.791
BMI (kg/m²)	98	29.2 ± 4.7	29.2 ± 4.2	29.3 ± 5.3	28.9 ± 4.9	0.791
Rel FAT (%)	85	30.0 ± 8.4	30.9 ± 7.1	31.5 ± 8.3	27.6 ± 9.8	0.158
Rel LTM (%)	85	58.0 ± 11.5	57.4 ± 10.2	56.1 ± 10.6	60.6 ± 13.7	0.329
Serum creatinine concentration (mg/dL)	101	1.9 (1.5-2.8)	1.5 (1.4-1.6)	1.9 (1.8–2.1)	3.4 (2.8–4.9)	**<0.001**
eGFR (mL/min/1.73 m²)	101	37 ± 14	52 ± 5	37 ± 4	19 ± 6	**<0.001**
Serum albumin concentration (g/dL)	99	4.4 (4.1–4.6)	4.6 (4.4–4.8)	4.4 (4.1–4.6)	4.3 (4.0–4.5)	**<0.001**
Serum glucose concentration (mg/dL)	100	98 (86–134)	90 (81–105)	107 (86–145)	101 (89–179)	**0.016**
HgbA1c (%)	101	5.8 (5.3–6.4)	5.5 (5.2–6.0)	6.2 (5.5–7.2)	5.9 (5.3–6.5)	0.056
HOMA-IR	100	3.8 (1.9–8.2)	3.8 (1.7–7.7)	5.1 (1.7–10.2)	3.4 (2.1–6.8)	0.653
Total cholesterol (mg/dL)	101	165.0 (142.0–207.0)	170.0 (143.0-234.0)	164.0 (144.3-204.5)	153.5 (129.5-184.0)	0.066
Low-density lipoprotein cholesterol (mg/dL)	101	101.0 (80.0–139.0)	119.0 (87.0–158.5)	97.5 (87.0–130.3)	93.0 (70.3–119.5)	**0.017**
High-density lipoprotein cholesterol (mg/dL)	101	42.0 (35.5–54.5)	45.0 (37.0–55.0)	40.0 (35.3–54.5)	41.5 (33.3–54.5)	0.339
Triglycerides (mg/dL)	101	144 (107.5–220.0)	141.0 (99.0–232.0)	144.5 (109.3–228.8)	155.0 (106.5–222.0)	0.651
Hemoglobin (g/dL)	101	13.2 ± 1.9	14.4 ± 1.4	13.0 ± 1.8	12.1 ± 1.6	**<0.001**
Hematocrit (%)	101	39.2 ± 5.4	42.3 ± 4.1	38.8 ± 5.1	35.9 ± 4.9	**<0.001**
CRP (mg/dL)	100	0.2 (0.1–0.4)	0.2 (0.1–0.3)	0.2 (0.1–0.6)	0.3 (0.1–0.5)	0.195
Fibrinogen (mg/dL)	61	342.1 ± 90.5	301.3 ± 78.3	355.1 ± 94.1	370.4 ± 88.1	**0.012**
TNF-alpha (pg/mL]	101	4.4 (3.5–5.5)	4.1 (3.3–4.8)	4.4 (3.4–5.2)	5.3 (4.4–6.9)	**<0.001**
Leptin (ng/mL)	101	11.3 (5.4–24.1)	9.8 (4.8–21.9)	12.6 (5.8–23.2)	11.1 (5.4–25.9)	0.609
Parathormone (pg/mL)	101	117.2 (73.3–222.8)	83.4 (56.2–137.6)	111.8 (73.4–172.7)	302.1 (138.6–441.8)	**<0.001**
Systolic blood pressure (mm Hg)	97	130 (125–140)	130 (120–137)	130 (120–140)	140 (130–150)	**0.005**

CKD, chronic kidney disease; BMI, body mass index; rel FAT, relative fat; rel LTM, relative lean tissue mass; eGFR, estimated glomerular filtration rate; HgbA1c, hemoglobin A1c; HOMA-IR, homeostatic model assessment of insulin resistance; CRP, C-reactive protein; TNF-alpha, tumor necrosis factor alpha; *p*-values < 0.05 are marked in bold.

**Table 2:** Sclerostin correlations with clinical and biochemical parameters.

Parameter	Total (*n* = 101)		CKD 3A (*n* = 37)		CKD 3B (*n* = 32)		CKD 4–5 (*n* = 32)	
	rho	*p*	rho	*p*	rho	*p*	rho	*p*
Age (years)	0.274	**0.006**	0.620	**<0.001**	0.159	0.384	0.108	0.564
Weight (kg)	0.105	0.305	0.058	0.735	0.343	0.059	−0.015	0.939
BMI (kg/m²)	0.194	0.058	0.119	0.484	0.383	**0.033**	0.146	0.451
Rel FAT (%)	0.213	0.052	0.321	0.069	0.370	0.063	0.074	0.726
Rel LTM (%)	−0.261	**0.017**	−0.368	**0.035**	−0.420	**0.033**	−0.032	0.881
Serum creatinine concentration (mg/dL)	0.247	**0.013**	0.080	0.639	0.106	0.564	0.081	0.664
eGFR (mL/min/1.73 m²)	−0.287	**0.004**	−0.266	0.112	−0.097	0.599	−0.124	0.505
Serum albumin concentration (g/dL)	−0.115	0.259	−0.191	0.265	0.068	0.712	0.175	0.355
Serum glucose concentration (mg/dL)	0.283	**0.005**	0.302	0.073	0.287	0.111	0.034	0.856
HgbA1c (%)	0.490	**<0.001**	0.713	**<0.001**	0.295	0.101	0.373	**0.039**
HOMA-IR	0.091	0.368	−0.047	0.384	0.452	**0.009**	−0.128	0.494
Total cholesterol (mg/dL)	−0.156	0.120	−0.390	**0.017**	0.107	0.558	0.062	0.741
Low-density lipoprotein cholesterol (mg/dL)	−0.159	0.114	−0.335	**0.043**	0.067	0.717	0.005	0.977
High-density lipoprotein cholesterol (mg/dL)	−0.187	0.063	−0.097	0.568	−0.356	**0.046**	0.066	0.722
Triglycerides (mg/dL)	0.078	0.439	−0.269	0.108	0.433	**0.013**	0.122	0.512
Hemoglobin (g/dL)	−0.318	**0.001**	−0.357	**0.030**	−0.040	0.829	−0.088	0.639
Hematocrit (%)	−0.319	**0.001**	−0.274	0.100	−0.063	0.730	−0.153	0.411
CRP (mg/dL)	0.180	0.074	0.462	**0.005**	−0.064	0.728	−0.181	0.329
Fibrinogen (mg/dL)	0.298	**0.021**	0.499	**0.021**	−0.151	0.550	0.189	0.412
TNF-alpha (pg/mL)	0.187	0.063	0.283	0.089	0.067	0.717	−0.071	0.703
Leptin (ng/mL)	0.349	**0.001**	0.285	0.087	0.394	**0.026**	0.379	**0.035**
Parathormone (pg/mL)	0.245	**0.014**	0.328	**0.047**	0.047	0.797	0.047	0.803
Systolic blood pressure (mm Hg)	0.140	0.172	0.335	**0.043**	−0.036	0.847	−0.149	0.441

CKD, chronic kidney disease; BMI, body mass index; rel FAT, relative fat; rel LTM, relative lean tissue mass; eGFR, estimated glomerular filtration rate; HgbA1c, hemoglobin A1c; HOMA-IR, homeostatic model assessment of insulin resistance; CRP, C-reactive protein; TNF-alpha, tumor necrosis factor alpha; *p*-values < 0.05 are marked in bold.

**Table 3:** The association of sclerostin with chosen parameters in stepwise multivariate quantile regression.

Parameter	Univariate Analysis *			Multivariate Analysis *		
	Coefficient	*p*-Value	95% Conf. Interval	Coefficient	*p*-Value	95% Conf. Interval
HgbA1c ≥ 6.5 (%)	221.20	**<0.001**	122.96–319.45	207.77	**<0.001**	126.76–288.78
Hemoglobin < 11 (g/dL)	184.92	**0.024**	25.40–344.45	x	x	x
Leptin (ng/mL)	6.35	**<0.001**	3.56–9.14	3.57	**0.003**	1.23–5.91
Parathormone (pg/mL)	0.06	0.793	−0.40–0.52	x	x	x
Age (years)	4.91	0.092	−0.82–10.64	6.50	**<0.001**	3.25–9.74
Rel LTM (%)	−3.44	**0.009**	−5.99–0.89	x	x	x
TNF-alpha (pg/mL)	2.50	0.687	−9.76–14.76	x	x	x

* adjusted for eGFR. HgbA1c, hemoglobin A1c; rel LTM, relative lean tissue mass; TNF-alpha, tumor necrosis factor alpha; *p*-values < 0.05 are marked in bold; x, placed for variables removed automatically from the model.

**Table 4:** Leptin correlations with clinical and biochemical parameters.

Parameter	Total (*n* = 101)		CKD 3A (*n* = 37)		CKD 3B (*n* = 32)		CKD 4–5 (*n* = 32)	
	rho	*p*	rho	*p*	rho	*p*	rho	*P*
Age (years)	0.103	0.303	0.103	0.543	0.112	0.542	0.043	0.813
Weight (kg)	0.631	**<0.001**	0.656	**<0.001**	0.652	**<0.001**	0.631	**<0.001**
BMI (kg/m²)	0.686	**<0.001**	0.685	**<0.001**	0.675	**<0.001**	0.675	**<0.001**
Rel FAT (%)	0.584	**<0.001**	0.572	**<0.001**	0.591	**0.001**	0.551	**0.004**
Rel LTM (%)	−0.559	**<0.001**	−0.535	**0.001**	−0.579	**0.002**	−0.534	**0.005**
Serum creatinine concentration (mg/dL)	0.081	0.421	0.182	0.280	−0.005	0.978	0.059	0.749
eGFR (mL/min/1.73 m²)	−0.078	0.441	−0.209	0.214	0.008	0.964	−0.038	0.837
Serum albumin concentration (g/dL)	0.065	0.521	0.165	0.338	−0.044	0.811	0.195	0.292
Serum glucose concentration (mg/dL)	0.033	0.747	0.115	0.505	0.161	0.377	−0.195	0.285
HgbA1c (%)	0.128	0.202	0.116	0.494	0.283	0.117	−0.021	0.911
HOMA-IR	0.349	**<0.001**	0.184	0.284	0.466	**0.007**	0.362	**0.042**
Total cholesterol (mg/dL)	−0.124	0.218	0.005	0.977	−0.182	0.318	−0.149	0.417
Low-density lipoprotein cholesterol (mg/dL)	−0.066	0.512	0.042	0.805	−0.053	0.774	−0.140	0.444
High-density lipoprotein cholesterol (mg/dL)	−0.455	**<0.001**	−0.385	**0.019**	−0.549	**0.001**	−0.411	**0.019**
Triglycerides (mg/dL)	0.343	**<0.001**	0.181	0.283	0.354	**0.047**	0.499	**0.004**
Hemoglobin (g/dL)	−0.012	0.903	−0.206	0.221	0.179	0.326	−0.021	0.908
Hematocrit (%)	0.001	0.992	−0.127	0.454	0.203	0.266	−0.048	0.795
CRP (mg/dL)	0.208	**0.038**	0.262	0.123	0.256	0.157	0.094	0.608
Fibrinogen (mg/dL)	0.366	**0.004**	0.326	0.149	0.276	0.268	0.311	0.159
TNF-alpha (pg/mL)	0.263	**0.008**	0.184	0.275	0.353	**0.047**	0.181	0.321
Parathormone (pg/mL)	0.106	0.291	0.211	0.211	0.079	0.667	0.084	0.646
Systolic blood pressure (mm Hg)	0.025	0.804	0.204	0.227	−0.011	0.952	−0.338	0.068

CKD, chronic kidney disease; BMI, body mass index; rel FAT, relative fat; rel LTM, relative lean tissue mass; eGFR, estimated glomerular filtration rate; HgbA1c, hemoglobin A1c; HOMA-IR, homeostatic model assessment of insulin resistance; CRP, C-reactive protein; TNF-alpha, tumor necrosis factor alpha; *p*-values < 0.05 are marked in bold.

## Data Availability

All relevant data analyzed during the current study are included in the article. Access to raw datasets may be provided upon reasonable request to the corresponding author following permission by the Ethics Committee and the Institute at which the study was conducted.
